# A Prominent Cell Manipulation Technique in BioMEMS: Dielectrophoresis

**DOI:** 10.3390/mi11110990

**Published:** 2020-11-03

**Authors:** Zeynep Çağlayan, Yağmur Demircan Yalçın, Haluk Külah

**Affiliations:** 1 Department of Electrical and Electronics Engineering, Middle East Technical University, Ankara 06800, Turkey; zcaglayan@mems.metu.edu.tr (Z.Ç.); y.demircan.yalcin@tue.nl (Y.D.Y.); 2 METU MEMS Research and Application Center, Ankara 06800, Turkey; 3 Mikro Biyosistemler Electronics Inc., Ankara 06530, Turkey

**Keywords:** biochip, biomems, biosensors, cancer cells, diagnostics, dielectrophoresis, lab-on-a-chip, marker-free particle manipulation, multidrug resistance, point-of-care

## Abstract

BioMEMS, the biological and biomedical applications of micro-electro-mechanical systems (MEMS), has attracted considerable attention in recent years and has found widespread applications in disease detection, advanced diagnosis, therapy, drug delivery, implantable devices, and tissue engineering. One of the most essential and leading goals of the BioMEMS and biosensor technologies is to develop point-of-care (POC) testing systems to perform rapid prognostic or diagnostic tests at a patient site with high accuracy. Manipulation of particles in the analyte of interest is a vital task for POC and biosensor platforms. Dielectrophoresis (DEP), the induced movement of particles in a non-uniform electrical field due to polarization effects, is an accurate, fast, low-cost, and marker-free manipulation technique. It has been indicated as a promising method to characterize, isolate, transport, and trap various particles. The aim of this review is to provide fundamental theory and principles of DEP technique, to explain its importance for the BioMEMS and biosensor fields with detailed references to readers, and to identify and exemplify the application areas in biosensors and POC devices. Finally, the challenges faced in DEP-based systems and the future prospects are discussed.

## 1. Introduction 

Micro-electro-mechanical systems (MEMS), presented as an extended branch of the conventional semiconductor very-large-scale-integration (VLSI) technologies, are small length scale devices and structures that integrate mechanical and electrical elements [1,2]. The dimensions of the MEMS devices or systems vary between micrometers to millimeters and the sizes of the MEMS components are in the level of micrometers [3,4]. Similar microfabrication techniques as the ones applied to produce integrated circuits (ICs) are followed to create MEMS structures and devices, indicating that as an innovative technology MEMS was evolved from semiconductor IC manufacturing [3,5]. The field of MEMS both in industry and academia has been accelerated in the 1980s and 1990s thanks to revolutionary developments achieved in new microfabrication processes derived from semiconductor IC manufacturing [6]. Desired complex microelectronic structures and features in MEMS devices are formed by following sequences of high-performance microfabrication techniques including mainly lithography, material deposition, patterning, and selective etching processes on a silicon and other substrates in a layer-by-layer manner [7,8]. Macro-scale effects are attained with MEMS devices thanks to their sensing, controlling, and actuating capabilities on the micro-scale [9]. Manufacturing and operating/functioning in small scale have enabled MEMS with advantages, such as superior performance, high integration, miniaturization, low power consumption, low cost, and high throughput [4]. The outstanding success attained in the MEMS technology has provided it to be applied in many different fields ranging across electronics, data storage, telecommunications, automotive industry, environmental monitoring, displays, biology, and chemistry [7,8,10,11,12,13].

Among other fields developed by MEMS technology, one of the most exciting and attractive research and application areas of MEMS is biological (or biomedical) micro-electro-mechanical systems, abbreviated as BioMEMS. BioMEMS can be referred as the MEMS-based devices or systems adapted for biological applications to process, manipulate, deliver, construct, or analyze biological entities [1,14,15]. Inherent advantages of MEMS technology such as working with small volume of samples, ease of manufacturing of portable and compact devices, achieving higher performance and sensitivity, providing small analysis times, being already compatible in size with organelles and cells, enabling reproducibility, and allowing rapid mass production with low cost make it well suited to biological and/or biomedical applications. BioMEMS apply traditional MEMS microfabrication techniques with some modifications and adaptations to manufacture ultra-small BioMEMS devices [16]. Micromachining, photolithography, soft lithography, and micromolding, as a mass-fabrication method, are the most extensively used techniques in the manufacturing of BioMEMS devices [17]. The materials used during the fabrication of BioMEMS devices can be divided into three main groups [14,15]. The first group contains the microelectronics and MEMS related materials such as silicon and glass [18,19,20]. The second group includes polymers and plastic materials such as polymethyl methacrylate (PMMA), polydimethyl siloxane (PDMS), and SU-8 [18,21,22,23]. The third group includes biological materials, such as proteins, cells, and tissues [14]. The first group of materials is well-established MEMS materials and has been widely used since the development of early MEMS devices, while the third group of materials is relatively new and provides promising opportunities for BioMEMS. The second group of materials is appealing since they offer several advantages such as biocompatibility, a crucial property that should be considered in BioMEMS and related devices, low electrical and thermal conductivities, reduced cost, reduced time, ease of fabrication, surface modification, and quick prototyping [24,25,26]. 

During the last decades, BioMEMS has gained significant attention and has grown drastically, both in terms of research and industry, by finding various biological applications including mainly detection of diseases, advanced diagnosis, therapy and treatment, drug-delivery, implantable devices, and tissue-engineering [1,14,27,28,29]. Nowadays, a wide range of digital biomedical devices that have been developed to measure and monitor different aspects of human behavior and performance are found on the market [30]. In this context, the medical industry, healthcare, patient monitoring, and healthcare management are among the most attractive subsections of BioMEMS, presenting tremendous growth and the development of smart point-of-care (POC) devices, biochips, and biosensors for treatment and diagnostics purposes. By definition, a biosensor is an analytical device that is designed to identify selectively and quantitatively one or more specific entities or analytes of biological interest by converting biological recognition action into a measurable signal [31,32]. Biosensors are innovative and powerful tools that provide accurate, real-time, quick, and reliable data for the analyte of biological interest together with being easy-to-operate, portable, and sensitive [33,34]. The field of biosensing has significantly evolved with the advancements achieved in fabricating miniature devices and sensing technology through BioMEMS, which in turn made BioMEMS as an indispensable and inseparable component of biosensor technology. Due to the advantages of BioMEMS for biological applications and biosensing, biosensors have found numerous applications in medicine and healthcare, including drug development, bioimaging and biosensing, environmental monitoring, and disease diagnosis, such as detection of genetic disorders, cancer, and pathogens alike [35,36,37,38,39,40,41]. 

One of the most essential and leading goals of the BioMEMS and biosensor technologies is to develop point-of-care (POC) testing systems. The POC testing is a prognostic or diagnostic test performed at a patient site to provide rapid results regarding diagnosis and treatment, eliminating the need for laboratories equipped with complex and expensive devices, and trained medical staff [42,43,44]. Performing analysis rapidly with high accuracy, and being easy-to-use with a sample-to-answer format are the most important requirements of POC systems [45]. These rigorous requirements of POC systems in disease diagnosis and therapy monitoring introduce new challenges for biosensor technology, such as identifying the analyte of interest in a very small sample volume with high selectivity and sensitivity, and integrating different components into a single platform [46]. In this context, there is a great need for reusable, robust, portable, sensitive, and cost-effective miniaturized biosensor platforms for POC applications. As an answer to this requirement, two important concepts in the biosensor and BioMEMS fields, namely lab-on-a-chip (LOC) and μTAS (micro Total Analysis Systems), have been developed. LOC and μTAS devices or systems are used to identify, detect, quantify, and isolate biological molecules. LOC devices aim to perform one to multiple conventional laboratory functions on a single miniaturized device for clinical and biological analysis [47,48,49,50]. This aim is accomplished essentially by taking the advantages of microfluidics [45,51,52,53,54]. Microfluidics technology works on the control and manipulation of liquids with extremely small volumes (in the range of 10^−9^ to 10^−18^ L) in a combination of microfluidic operational units such as microchannels, microchambers, and microvalves [15,55]. Microfluidic devices offer a high surface area to volume ratio that enables portability, which is an essential requirement for POC testing [56]. At the same time, this unique property of microfluidics, namely the high surface area to volume ratio, significantly shortens the analysis time and provides fast results that are crucial for POC applications [45]. Microfluidics technology is also present in μTAS, which is another attractive branch for POC testing [57]. μTAS, as a parallel term to the LOC, can be defined as the miniaturization of the entire sequence of laboratory functions required to perform chemical analysis in a single chip [58,59,60]. To achieve this goal, these systems integrate several steps of chemical analysis processes such as sample pre-treatment, sample preparation, injection, dilution, separation, reaction, filtration, and detection via employing micropumps, microvalves, switches, and mixers [57,61,62]. Integration with microfluidics and small-scale operation of LOC and μTAS instruments provides significant advantages, including efficient cell, molecule, and particle isolation and immobilization; smaller volume of sample and carrier usage; low reagent consumption; providing advanced transportation mechanisms; and high capability of integration of different parts such as mixers, micropumps, reaction chambers, separators, electrodes, channels, and detectors into a single device [63]. 

To achieve the aforementioned advantages and to realize POC devices in medical treatment, diagnostics and related applications, the manipulation of the bioparticle (i.e., cell, bacteria, virus, DNA, and protein) in the analyte of interest is a vital task for the LOC, μTAS, and biosensor platforms. Therefore, particle manipulation, which is essentially the ability to control the motion of particles and the fluid environment around them, has been extensively studied. The methods proposed and developed for the manipulation of particles in microfluidic systems can be grouped as active and passive methods determined according to the presence of external force fields integrated into the system [64,65,66,67]. Passive methods do not use external forces to control the movement of particles, instead they merely depend on the channel geometry, and the interactions between particles, fluid flow, and microchannel topology [68,69,70]. Although passive manipulation methods can be performed with simpler structures and allow higher flow rates, their application is limited due to their dependence on fixed microchannel design and geometry [66,67]. These methods are more preferred when input energy is critical [64,70]. In active methods, different forms of fields are employed to generate the external force required for particle manipulation. Active methods provide relatively better throughput and efficiency together with more precise and real-time control over the particles [64,67]. Magnetic, optic, acoustic, and dielectrophoretic fields and related principles are utilized as the source of external forces in most of the active manipulation methods. In magnetic-based methods, a magnetic field, generated either via permanent magnets or electromagnets, is applied to manipulate inherently magnetic and/or magnetically labeled particles [71,72,73,74]. Although magnetic-based methods provide high specificity, low particle damage, and fast operation, the inability to naturally manipulate most of the particles by the magnetic field poses a major challenge for these systems and consequently requires expensive and time-consuming labeling procedures in related applications [64,65,66]. In optical manipulation, the motion of the particles is precisely controlled in a contactless manner by the optical forces resulting from the net momentum change between the incident beam of light and the light scattered from the targeted particle [75,76,77,78,79]. However, these methods require complex and expensive equipment to perform optical operations in addition to some concerns regarding heating and possible bioparticle damage [80,81]. In acoustic-based methods, the acoustic radiation force created by ultrasonic standing waves is used to provide rapid, contactless, and label-free manipulation of particles without affecting their viability [82,83,84,85]. In order to efficiently transmit acoustic power to the fluid and to achieve successful operation in acoustic-based systems, material selection for the device and precise design of microchannel geometry are extremely critical [65,86]. 

The electric field, another source field used in active manipulation techniques, is applied to control particle movements through electrokinetic mechanisms [75,81,87]. Dielectrophoresis (DEP), one of the electrical manipulation techniques, is the movement of particles in a non-uniform electrical field due to polarization effects [88]. When compared to other available methods, DEP is one of the most attractive and promising manipulation techniques due to its prominent advantages [89,90,91]. DEP is based on intrinsic dielectric properties which are determined by the morphological, structural, and chemical state of the particle [92,93,94]. Therefore, DEP is a label-free technique. It enables simplification of preparations steps [89,91,95,96]. DEP is a non-destructive and non-invasive technique that removes concerns regarding cell viability and alterations in cell function [90,97,98]. This allows performing consecutive processing steps, further investigation and downstream analysis on the manipulated particles [99,100]. In addition, thanks to improvements in microfabrication technologies, electrode arrangement, one of the most important factors in achieving effective DEP operation, can be easily applied into chips and microfluidics, showing that the DEP technique is also perfectly compatible for equipment miniaturization [101,102]. Therefore, DEP presents great applicability in LOC and biosensor fields. DEP is also advantageous because of its accuracy, low-cost, efficiency, sensitivity, selectivity, being rapid, consuming low samples and reagents, providing precise manipulation, operating easily, and enabling direct electronic interface [89,101]. Due to all these prominent advantages, DEP has become an indispensable particle manipulation technique in microchips developed for biomedical applications [91,101,103]. Consequently, DEP has been applied to control the motion of various bioparticles such as cells, including diseased cells [104,105,106], healthy cells [107,108,109], stem cells [98,110], microorganisms [111,112,113,114,115,116], proteins [117,118,119], DNA [120,121,122], and exosomes [123]. 

DEP, a prominent particle manipulation technique, has been extensively studied by many research groups and scientists in biosensor and POC applications. In this framework, numerous successful DEP-based systems have been established. The intended scope of the article is not to discuss in-depth and comprehensively all of the numerous DEP-based biosensors and POC applications reported. Instead, we aim to provide a general introduction to the DEP technique, to explain its importance for the BioMEMS and biosensor fields by providing detailed references to readers, and to identify and exemplify the application areas in biosensors, LOC, and POC devices based primarily on the studies carried out in our group, BioMEMS Research Group, Middle East Technical University. The fundamental theory and principles of DEP will be described in Section 2. Applications of DEP in POC and biosensor environments, given the research headings of our organization, namely: (i) Dielectric cell characterization, (ii) multidrug resistance (MDR) research, (iii) cell separation, and (iv) DEP integration in biosensors for other purposes, will be discussed in Section 3. Finally, the challenges faced in DEP-based systems and possible solutions to solve these challenges, and the future perspectives of the DEP technology in POC testing and biosensor applications will be covered in Section 4.

## 2. DEP Background 

DEP is an electrokinetic phenomenon that refers to the motion of dielectric particles in a non-uniform electric field [88,124,125]. One of the unique features of this technique is that it can be applied to both neutral and charged particles. When a particle is placed in a non-uniform electric field, a lateral force is exerted on it due to the electrical polarizability differences between the particle and the medium in which it is suspended. This force is called as dielectrophoretic (DEP) force. The DEP force exerted on a spherical particle is formulated as [126,127,128]

(1) $$F_{DEP}=2\pi R^{3}\varepsilon_{m}\varepsilon_{0}Re\left(f_{CM}\right)\nabla {\left|E\right|}^{2}$$

where R is the radius of the spherical particle, $\varepsilon_{m}$ is the relative permittivity of the surrounding medium, $\varepsilon_{0}$ is the permittivity of the vacuum, ∇ shows the gradient operation, and E is the amplitude of the electric field. $Re\left(f_{CM}\right)$ term represents the real part of the Clausius–Mossotti (CM) factor. The CM factor ($f_{CM}$) is expressed by

(2) $$f_{CM}=\frac{\varepsilon_{p}^{*}-\varepsilon_{m}^{*}}{\varepsilon_{p}^{*}+2\varepsilon_{m}^{*}}$$

where $\varepsilon_{p}^{*}$ is the complex permittivity of the particle and $\varepsilon_{m}^{*}$ is the complex permittivity of the surrounding medium. $\varepsilon_{p}^{*}$ and $\varepsilon_{m}^{*}$ are defined as

(3) $$\varepsilon_{p}^{*}=\varepsilon_{p}\varepsilon_{0}-j\frac{\sigma_{p}}{w}$$

(4) $$\varepsilon_{m}^{*}=\varepsilon_{m}\varepsilon_{0}-j\frac{\sigma_{m}}{w}.$$

Complex permittivity depends on the material permittivity (ε) and material conductivity (σ), where subscripts “*p*” and “*m*” represents the particle and the medium, respectively. The imaginary number ($\sqrt{-1}$) is indicated with j term and w represents the angular frequency (w=2πf), which relates the $Re\left(f_{CM}\right)$ term to the frequency of the applied electric field. 

The polarizability parameter $Re\left(f_{CM}\right)$ describes the relationship between the $\varepsilon_{p}^{*}$ and $\varepsilon_{m}^{*}$, and changes as a function of the applied field frequency. Polarizability shows the ability of a material to respond to an electric field. It is a measure of a material’s ability to generate charge at the interface [102]. When a particle placed in a medium is exposed to an electric field, the charges within both the particle and the surrounding medium will be rearranged at the interface between the particle and the medium. Charge accumulation produces an induced electric dipole indicating that different charge densities are present on either side of the particle. In the presence of a non-uniform external electric field, different forces will be exerted from each side of the particle. As a result, a net force called the DEP force will be generated, resulting in particle motion. Induced charge accumulation, known as polarization, and the resultant DEP force depend on the polarizability of the particle and the medium [102,129,130]. Particles with higher polarizability than the surrounding fluid are attracted toward the higher electric field strengths, which is defined as positive DEP (pDEP). Particles with lower polarizability than the surrounding fluid are pushed toward lower electric field strengths, which is defined as negative DEP (nDEP). Note that, $\varepsilon_{p}^{*}>\varepsilon_{m}^{*}$ implies that $Re\left(f_{CM}\right)$ is positive ($Re\left(f_{CM}\right)>0$) in pDEP. Additionally, $\varepsilon_{p}^{*}<\varepsilon_{m}^{*}$ implies that $Re\left(f_{CM}\right)$ is negative ($Re\left(f_{CM}\right)<0$) in nDEP. Theoretically, $Re\left(f_{CM}\right)$ term varies between −0.5 and +1.0 [90]. The frequency point at which transition from nDEP to pDEP (or pDEP to nDEP) occurs is defined as crossover frequency [131]. At crossover frequency, the net DEP force acting on the particle is equal to zero. At this frequency, the complex permittivity of the particle and the surrounding medium are exactly equal [102]. The basic DEP theory shows that in the uniform electric field (∇E term is zero) the DEP force acting on the particle will be zero. In addition, the DEP force depends on the particle size, in other words, the DEP force is ponderomotive; as a result, there will be more DEP force for larger particles when all other factors remain the same [126]. 

To represent the cells theoretically, the multi-shell model or the single-shell model is used, determined according to the complexity of the particle. The single-shell model, the simplest particle modeling, treats the cell cytoplasm as a homogeneous sphere covered with a thin cell membrane. This model replaces the real two-layered (cell membrane and cytoplasm) particle with a homogeneous sphere with an effective complex permittivity [102,126,127]. In the single-shell model, the effective complex permittivity $\varepsilon_{p}^{*}$ is described as

(5) $$\varepsilon_{p}^{*}=\varepsilon_{mem}^{*}\left[\frac{{\left(R/\left[R-d_{mem}\right]\right)}^{3}+2\left(\frac{\varepsilon_{cyt}^{*}-\varepsilon_{mem}^{*}}{\varepsilon_{cyt}^{*}+2\varepsilon_{mem}^{*}}\right)}{{\left(R/\left[R-d_{mem}\right]\right)}^{3}-\left(\frac{\varepsilon_{cyt}^{*}-\varepsilon_{mem}^{*}}{\varepsilon_{cyt}^{*}+2\varepsilon_{mem}^{*}}\right)}\right]$$

where $d_{mem}$ is the membrane thickness, R is the outer radius of the particle, $\varepsilon_{cyt}^{*}$ is the complex permittivity of the cytoplasm, and $\varepsilon_{mem}^{*}$ is the complex permittivity of the membrane. The effective complex permittivity $\varepsilon_{p}^{*}$ is inserted into Equation (2) to obtain the CM function. Most particles are complex and heterogeneous as they consist of nuclei, cytoplasm, and cell membrane with different electrical properties [101]. Therefore, to accurately represent their heterogeneous structures the single-shell model can be extended to the multi-shell model. For example, erythrocytes can be represented with a single-shell model. However, modeling of leukocytes that include nucleus requires a three-shell model in which the plasma membrane, cytoplasm, and membrane that covers the nucleoplasm are presented with three different shells [126]. Moreover, plant cells and many single cell microorganisms (e.g., bacteria and yeast cells) are typical examples of walled structures that can be represented with multi-shell model to reflect their structural complexity [127]. 

The electric field gradient is the most essential requirement of the DEP technique. As given in Equation (1), induced DEP force on the particle of interest depends on the electric field gradient. The required non-uniform electric field is generated by the electrodes. The geometry and distribution of the electrodes, the materials used for the electrodes, and the fabrication steps followed in their production are decisive parameters for the generated non-uniform electric field and the DEP force [80,89,90,126]. Electrode configuration must be optimized to achieve an efficient DEP operation. Many different electrode geometries and arrangements have been implemented in DEP-based systems. The 2D planar or 3D microelectrodes are mainly used to create a non-uniform electric field. Alternatively, insulator structures embedded in the microchannel are also utilized to attain electric field gradient. The microelectrode configurations frequently used in DEP-based systems can be listed as follows: Polynomial, slanted, parallel or interdigitated, top–bottom patterned, quadrupole, curved, microwell, oblique, matrix, castellated, sidewall patterned, extruded, insulator-based or electrodeless, and contactless [89,90,126,132,133,134]. Various types of materials including metals (i.e., gold, chromium, and platinum), carbon, conductive polymer composites, and silicon are employed to fabricate electrodes [89,132]. In the case of insulator structures, glass, insulating polymers, and oil are used [132]. Advances in microfabrication techniques achieved with MEMS technology have allowed electrode structures to shrink dramatically and be manufactured close to each other. As a result, much more complex electrode arrangements with any arbitrary geometry were fabricated and the voltage applied to generate the required DEP force was decreased significantly [125,135]. Various basic microfabrication techniques including photolithography, reactive ion etching, injection molding, thin-film deposition, conventional machining, hot embossing, soft lithography, wet etching, electroplating, and substrate bonding are followed during the manufacturing of microelectrodes and DEP microdevices [91,132,135]. Finally, DEP allows different operating strategies to improve performance in specific applications. These strategies determine and operation mechanisms and how the generated forces interact with each other in the manipulation of particles. These operating strategies can be grouped as: Gravitational field-flow-fraction (FFF), traveling wave, lateral sorting, multi-step, electrothermal-assisted, barrier-assisted, pulsed, multiple frequency DEP, immuno-assisted sorting, medium conductivity gradient, light-induced, marker-specific, and electrorotation [133,134].

## 3. Applications of DEP 

Here, we review and discuss recently reported DEP-based applications in BioMEMS and biosensor fields, which are categorized based primarily on the studies carried out in our group, BioMEMS Research Group, Middle East Technical University.

### 3.1. Dielectric Characterization of Cells 

Analysis and characterization of particles according to their intrinsic dielectric properties are very critical for biosensor platforms in medical treatment, diagnosis, and related biomedical applications. Impedance spectroscopy and AC electrokinetic techniques, mainly DEP, are two dominant ways used to determine the dielectric properties of particles [136]. Although impedance spectroscopy provides label-free measurements, it has fundamental limitations such as time-consuming and low efficiency [136,137]. Moreover, the accuracy in the determination of electrical parameters is greatly affected by microchannel geometry, cell capture mechanism, electrode size, particle volume, and particle–particle interactions [137,138]. At this point, DEP shows great potential for the electrophysiological characterization of particles without any physical contact. DEP characterization involves the determination of dielectric properties of particles by analyzing the response of one or more particles to the electric field induced forces as a function of time, location, or frequency of the applied field [139,140,141]. The dielectric properties of particles are governed by the size, distribution of surface charges, morphology, and intracellular and extracellular events [93,94,142,143]. Consequently, the dielectric properties of particles are used to analyze changes in particles’ electrophysiological parameters and give valuable insight into their physiological state. In addition, the dielectric properties of particles are extensively used in the development of DEP-based manipulation systems. 

**Figure 1:**
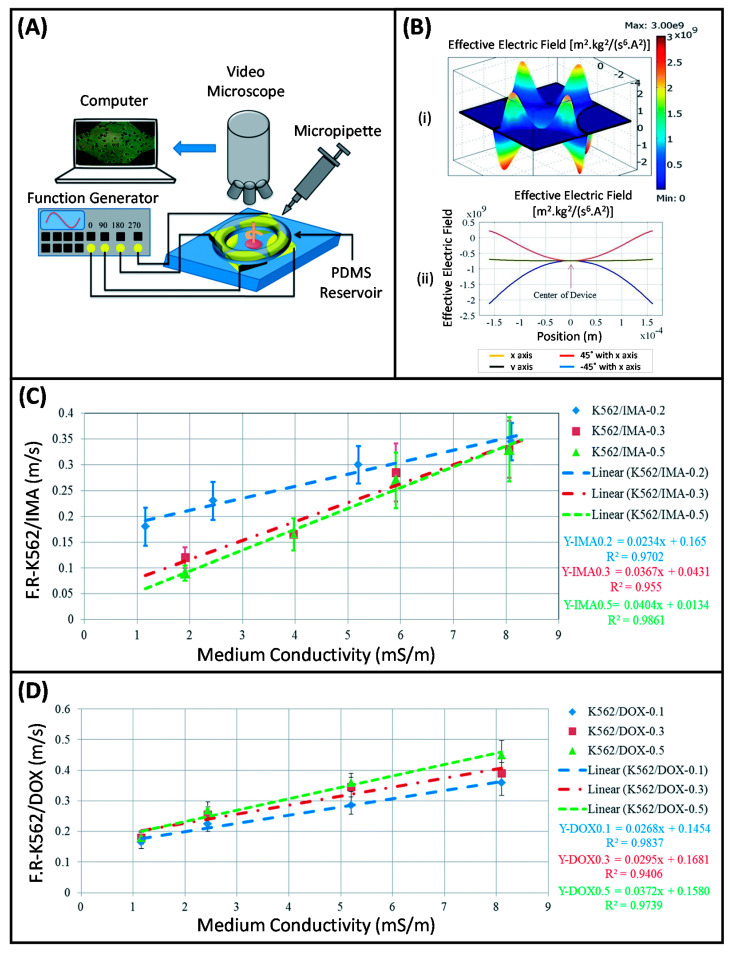
(**A**) The experimental setup for electrorotation (ER) tests. (**B**) Simulation results were performed to examine the effective electric field (EEF) distribution on the device surface. (**i**) Height representation of the EEF distribution. (**ii**) The variations of the EEF distribution along four lines passing through the electrode center. (**C**) Straight line fitted to the data points of the peak rotation frequency of K562/IMA cells and cell radius versus medium conductivity. (**D**) Straight line fitted to the data points of the peak rotation frequency of K562/DOX cells and cell radius versus medium conductivity. Reproduced from [160] with permission from The Royal Society of Chemistry.

**Figure 2:**
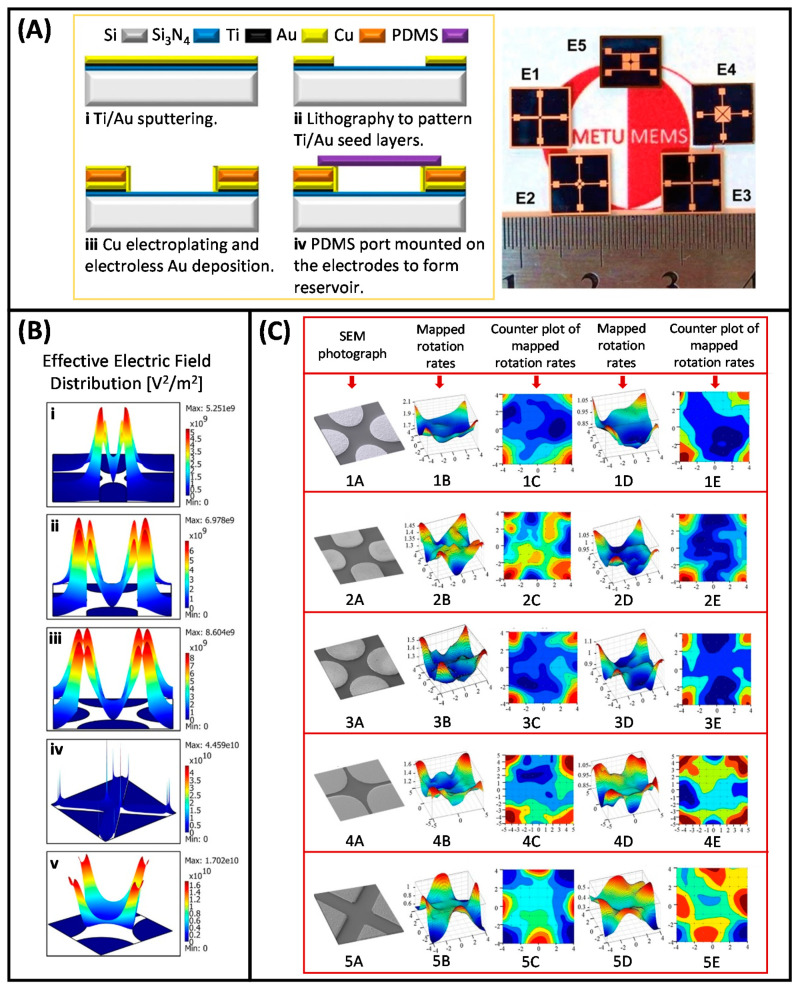
(**A**) Single mask fabrication process flow of the ER devices (**i**) Ti/Au sputtering, (**ii**) lithography, (**iii**) Cu electroplating, and (**iv**) mounting polydimethyl siloxane (PDMS) port, with the photograph of fabricated prototypes. (E1) Oblate elliptical, (E2) circular, (E3) prolate elliptical, (E4) triangular, and (E5) polynomial electrodes. (**B**) The height presentation of the EEF distributions for each electrode geometry, (**i**) prolate elliptical, (**ii**) oblate elliptical, (**iii**) circular, (**iv**) polynomial, and (**v**) triangular electrodes. (**C**) The extracted rotational torque maps together with SEM images of the ER devices with prolate elliptical (**1A**–**E**), oblate elliptical (**2A**–**E**), circular (**3A**–**E**), polynomial (**4A**–**E**), and triangular (**5A**–**E**) electrodes [162]. Copyright 2015, John Wiley and Sons.

Electrorotation (ER), one of the operating strategies of DEP, is commonly utilized to examine the electrical properties of particles. In the ER technique, a rotating electric field is used to induce controlled rotation in the polarizable particles [127,131]. The frequency dependent rotational velocity, which is the parameter of interest in ER, is used to effectively and accurately extract the particle interior and surface dielectric properties [137,144,145]. This technique has been applied to characterize many types of particles such as leukocytes [138,146,147,148], red blood cells (RBCs) [149,150], platelets [151], fibroblasts [152], yeast cells [153], various cancer cells [138,154,155,156], and bacteria [157]. In order to gain detailed information about the biophysiological states and dielectric properties of the cells, and to determine their DEP-induced responses in DEP-based manipulation systems to be developed later, our group has started to work with the ER technique. In this context, MEMS-based ER studies were initiated by analyzing the dielectric properties of imatinib- and doxorubicin-resistant K562 human leukemia cells [158,159]. Bahrieh et al. reported a microfabricated ER device with 3D polynomial electrodes to conduct dielectric characterization of sensitive and multidrug resistant (MDR) K6562 human leukemia cells (Figure 1A,B) [158,159,160]. MDR is a condition in which cancer cells show resistance to chemotherapeutic drugs with distinct chemical structures, and it is an important obstacle in cancer treatment performed with chemotherapy [161]. A more detailed explanation of MDR will be provided in Section 3.2. Dielectric properties of cytoplasm and membrane parts of wild type K562 cells and MDR K562 cells with different resistance levels to chemotherapeutic drugs, imatinib (IMA, 0.2–0.5 µM) and doxorubicin (DOX, 0.1–0.5 µM), were determined to analyze the relationship between the level of drug resistance and dielectric properties of the cells. In this study, straight lines fitted to the data points of the peak rotation frequency of the cells and cell radius versus medium conductivity were obtained. The dielectric parameters of the corresponding cell populations, namely the membrane capacitance and effective conductance, were extracted using the slope and intercept of the fitted line, respectively (Figure 1C,D). ER results showed that considerable changes occurred in the internal and membrane dielectric properties between subtypes of MDR K562 cells as the level of resistance to chemotherapeutic drugs varied. Although sensitive and MDR K562 cells had similar morphology and size, it was proven that the dielectric properties of these cells were highly affected by the level of drug resistance. The variations detected in DEP crossover frequencies and dielectric properties for sensitive and MDR K562 leukemic cells also indicated that the corresponding cells could be separated by using DEP [160]. In a further study, to determine the optimum structure for ER, the examination of the electrical characteristics of ER devices with different electrode geometries by using both finite element modeling (FEM) and experimental analysis was reported [162]. ER devices with five extensively used electrode geometries, namely oblate elliptical, prolate elliptical, circular, triangular, and polynomial, were designed and fabricated with a single mask (Figure 2A). In-plane and out-of-plane distribution of rotational torque (ROT-T) and effective electric field (EEF) on the microfabricated ER devices were studied by using both FEM and experimental method (Figure 2B,C). The results of this study provided valuable information in terms of determining the design and implementation parameters to achieve higher accuracy in ER studies. 

**Figure 3:**
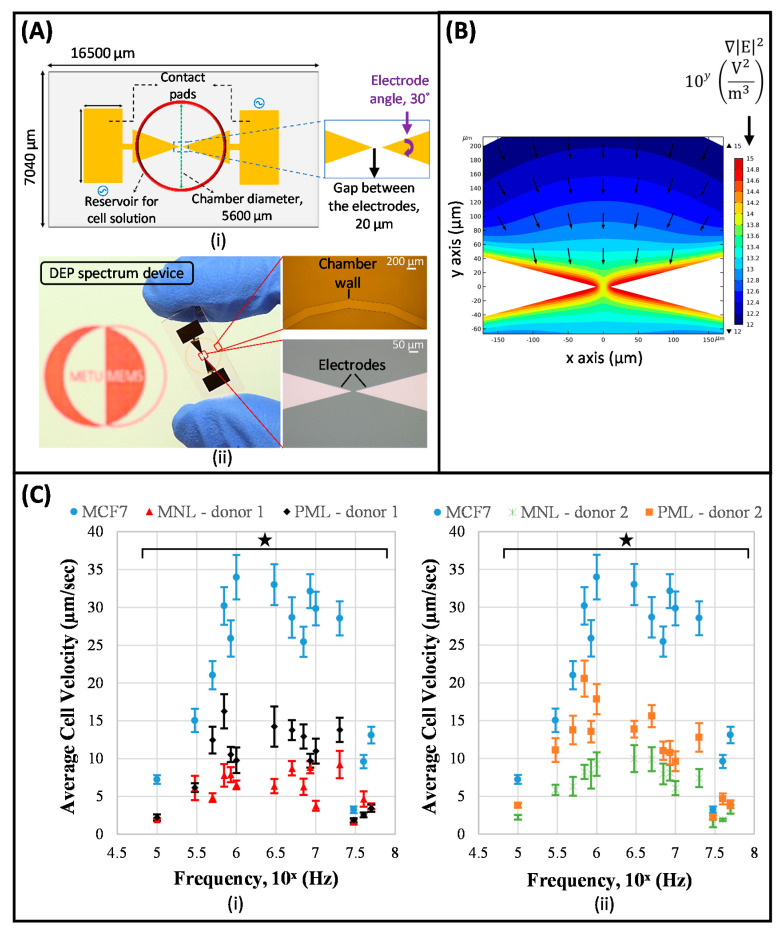
(**A**) The schematic illustration (**i**), and the photograph (**ii**) of the dielectrophoresis (DEP) spectrum device. (**B**) Simulation results presenting the direction and distribution of the generated electric field gradient on the device (due to the symmetry, only the first two quadrants are shown). (**C**) DEP spectra of MCF7 cells, polymorphonuclear and mononuclear leukocytes of two donors. (**i**) DEP spectra of MCF7 cells and leukocytes of donor 1: 32-year-old woman. (**ii**) DEP spectra of MCF7 cells and leukocytes of donor 2: 39-year-old woman. Mean values (symbols) and standard error of the means (error bars) are plotted for each examined frequency. Statistical significance (★ *p* < 0.05) between the MCF7 cells and leukocytes is represented for the examined frequency range [175]. Copyright 2020, John Wiley and Sons.

Applying a non-uniform electric field to induce a translational DEP force on the cells, i.e., application of regular DEP technique, is the other method to examine DEP characterization of cells. In this method, DEP crossover frequency determination and DEP spectrum analysis are the two typically applied approaches [139]. In DEP crossover frequency determination, crossover frequency of the cells is examined to determine electrical parameters of them by conducting numerical analysis and fitting to cell polarization model [163,164,165,166,167]. DEP spectrum analysis, which can be considered as an extended version of DEP crossover frequency determination, enables the DEP response of cells to be obtained directly across a wide frequency range [139,140]. In this approach, the levitation height, velocity, trapping voltage, collection or concentration of the cells, as a function of frequency, could be the parameter of interest [143,168,169,170,171,172,173]. Alshareef et al. studied DEP characteristics of MCF7 human breast cancer cells and HCT-116 colorectal cancer cells by correlating their DEP velocity to the induced DEP force on the corresponding cells [94]. DEP force acting on each cell type was quantified across AC electric frequency range for cell suspensions with different conductivities. Next, an ideal frequency region for the isolation of these two cell types was determined, and by employing this information, the isolation of the corresponding cells was accomplished. Labeed et al. examined the electrical parameters of K562 human leukemic cells and their MDR counterpart K562AR cells by using DEP collection spectrum data [161]. Here, the cell collection profile was obtained by counting the collected cells after 1 min exposure to DEP over the frequency interval between 10 kHz to 20 MHz, at five frequencies per decade. The electrical parameters of the corresponding cells were determined by fitting the measured average DEP collection spectrum to the single-shell model. Considering the importance of these studies, we added the DEP spectrum method to our research portfolio to attain a direct examination of the DEP responses of the cells across a wide frequency range. In this context, studies were initiated by examining the DEP spectrum of MCF7 human breast cancer cells [174]. Çağlayan et al. recently reported a DEP spectrum approach that performed the DEP characterization of cells by correlating the measured average velocity parameter to the induced DEP response [175]. The DEP spectra of human leukocyte subpopulations, mononuclear leukocytes and polymorphonuclear leukocytes, and MCF7 breast cancer cells were examined by this technique in the frequency range of 100 kHz–50 MHz by using microfabricated DEP spectrum device with reciprocal V-shaped planar electrodes (Figure 3A,B). The results showed that different DEP responses existed for mononuclear and polymorphonuclear leukocytes, as a result of morphological and cell surface property differences in leukocyte subpopulations. DEP spectrum of MCF7 cancer cells was significantly different from that of leukocytes, as expected since cancerous state brings more complex surface morphologies, and changes in surface charges and dielectric properties when compared with normal blood cells (Figure 3C) [175]. Especially, the strength of the DEP force exerted on MCF7 cells was higher between 850 kHz and 20 MHz. The presented system is a generic platform that offers great potential to directly analyze the DEP response of cells, determine whether and how well cell subpopulations can be isolated with DEP.

### 3.2. Multidrug Resistance (MDR) Detection in Cancer Cells 

MDR is a condition in which cancer cells develop simultaneous resistance to numerous structurally and functionally distinct drugs. Cancer patients cannot answer chemotherapy when MDR arises. Therefore, it is an impediment towards curative cancer treatment [176]. Almost half of the cancer patients suffer from MDR, showing either *de novo* resistance, prior to chemotherapy, or acquired resistance, arising due to the drug treatment [177,178]. Diagnosis of MDR is vital to choose the most appropriate treatment and to speed up the recovery period [179]. Although there exist different mechanisms behind MDR, one of the most abundant mechanism is the overexpression of membrane associated proteins, including P-glycoprotein (P-gp) and multidrug resistance associated protein (MRP-1). Therefore, conventional MDR diagnosis techniques, including in vivo imaging techniques, protein assays, and flow cytometry, are focused on the detection of these proteins [178,180]. These techniques have enough capability to detect MDR. However, they require highly experienced personnel. Additionally, they are not time- and cost-effective. Due to labeling procedures, which are the key elements utilized in these techniques, they are not suitable for frequent use during prognosis, because of the side effects. In order to provide MDR diagnosis effectively, a rapid, cost-effective, easy-to-use, and label-free method is necessary without conceding the sensitivity and specificity. 

**Figure 4:**
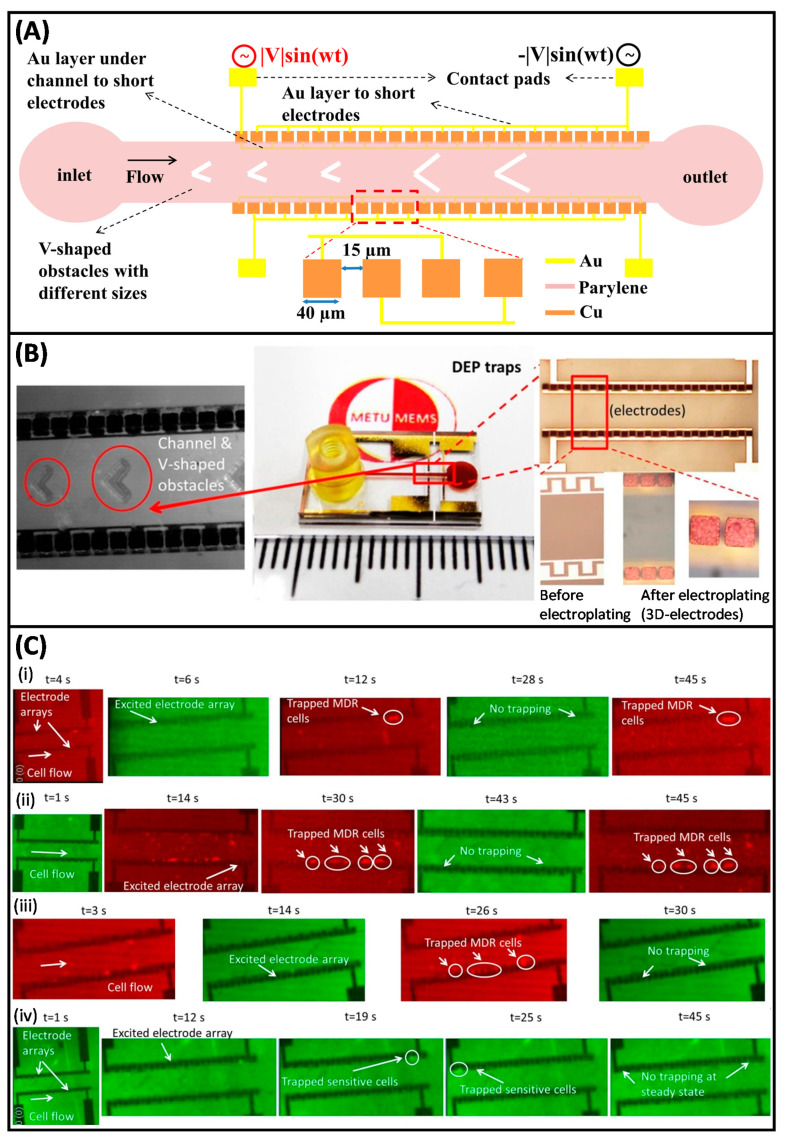
The schematic (**A**) and fabricated (**B**) version of the DEP-based multidrug resistance (MDR) detection device. (**C**) Pseudo colored screenshot images of trapped K562/doxR (**i**) and K562/imaR (**ii**) cells taken at a flow rate of 6.67 µL/min. (**iii**) The separation of K562/imaR cells and wild type K562 cells under 10 µL/min flow rate to make flow rate performance analysis. Working solution was the mixture of K562/imaR and wild type K562 cells with 2.5 × 10^3^ cells/mL and 2.5 × 10^5^ cells/mL, respectively (f = 48.64 MHz, V = 9 V_pp_). (**iv**) The screenshots taken during the analysis of wild type K562 cells only. No cell was trapped until t = 12 s. Some of the cells were trapped during the experiment. At the end of 45 s, required duration to reach steady state in channels, all cells were carried away [182]. Copyright 2015, John Wiley and Sons.

In last decades, it was reported that MDR created deviations in the biophysical properties of cancer cells, enabling label-free diagnosis of it, since MDR associated membrane proteins can also have the activity of ion channel modulation in cells [161,181,182,183,184]. Labeed et al. revealed that the cytoplasmic conductivity of doxorubicin resistant K562 (K562/doxR) cells was almost 2 times higher than that of drug sensitive K562 cells, as analyzed by DEP collection spectra [161]. They also correlated P-gp overexpression of cells with MDR in this study. Coley et al. reported that MCF7 cells and its MDR progenies (MCF7-TaxR, MCF7-DoxR, and MCF7-MDR1) had different cytoplasmic conductivities (MCF7-TaxR<MCF7<MCF7-MDR1<MCF7-DoxR) [185]. We started to study MDR detection in leukemia via DEP in 2010 in our group. In the following part, these studies are delivered by focusing on the importance of these contributions in terms of the execution of DEP in MDR detection. In the studies of Labeed et al., doxorubicin and K562 leukemia cell line were chosen as chemotherapeutic and cell type, respectively. By inspiring from these studies and using the electrical properties of K562/doxR cells obtained in these studies, we modeled imatinib resistant (imaR) and sensitive K562 cells to determine a crossover frequency value to sense the discrimination between these cells under continuous flow inside a microchannel. To achieve this purpose, a 3D-electrode contactless DEP device was designed and fabricated (Figure 4A,B). In this device, electrodes were insulated with a thin parylene layer (0.5 µm), reducing Joule heating and cell damaging without increasing the required voltage to manipulate cells by DEP. It was shown that this system achieved the trapping K562/imaR cells at a concentration of 6.25 × 10^5^/mL and 10 μL/min flow rate under 9 V_pp_ sinusoidal signal at 48.64 MHz. No trapping occurs for non-resistant K562 cells when the same experimental conditions were applied [186]. Next, we proved that the detection of imatinib (500 nM, imaR) and doxorubicin (500 nM, doxR) resistant K562 cells in a mixture, composed of MDR and wild type cells, was achievable with this platform at the previously determined frequency (48.64 MHz) [182]. In Figure 4(Ci,ii), the screenshots of the detection of K562/imaR and K562/doxR cells in the separation tests of MDR K562 and wild type cells at 6.67 µL/min flow rate through 45 s test period were presented, respectively. Pseudo colored images in green represented wild type cells while red ones were utilized for MDR cells in these figures. They were shown together to demonstrate that no trapping was observed for wild type K562 cells while MDR progenies were trapped in 3D electrodes. MDR detection was carried out at higher flow rates (10 µL/min). At this flow rate, 30 s test period of K562/imaR detection in a mixture, consisting of MDR and wild type K562 cells was displayed in Figure 4(Ciii). The device was also capable to detect MDR at this flow rate. At the beginning of this duration, some of the wild type cells were trapped. However, they were released at the end of duration (45 s) required for steady state although they have 100 times higher concentration than that of MDR cells in this mixture. For further confirmation, we repeated the analysis with only wild type K562 cells at 6.67 µL/min flow rate. Figure 4(Civ) presents the results at which trapping was obtained after 25 s while at the end of 45 s, all wild type cells were released from electrodes. 

We hypothesized that the DEP response of MDR cancer cells changed with the level of resistance since drug resistance is correlated with ion channel modulators in the cell membrane and ion channels directly affect the cell electrical properties [161]. To prove this hypothesis, we determined the trapped cell number on electrodes of our DEP device via light intensity analysis in Image J (Figure 4B). Since 3D-electrodes produce a uniform DEP force through the channel depth, cells can accumulate on top of each other. Therefore, for precise quantification of cells by the examination of light intensity, an algorithm was created, using cell transparency [187]. K562/doxR cells resistant to 100, 300, 500, and 1000 nM doxorubicin were utilized in this study. Trapped cell number was significantly increased up to 300 nM while further increase in the level of drug resistance was not observed in DEP response, significantly. This nonlinear connection between DEP response and drug resistance level might be interpreted that P-gp overexpression might not be the main cause of MDR at high drug concentrations and cells established another mechanism to resist drugs. Or, cytoplasmic conductivity value could not go beyond a point although P-gp level increased, since cell metabolism might be damaged by the unlimited increase in conductivity of cytoplasm. Verification of P-gp expression by biological techniques should be performed to resolve the reason behind this.

Finally, we offered a DEP-based LOC device, consisting of two consecutive DEP units, to detect MDR K562 cells in a cell mixture, having RBCs and MDR K562 cells [188]. First DEP unit was responsible for the depletion of RBCs and the second one was for the capturing of MDR K562 cells. The first unit achieved to eliminate RBCs up to 60% which was high enough to provide 100% selectivity in the second unit at which only MDR K562 cell trapping was achieved at a flow rate of 10 µL/min and at 20 V_pp_. On the other hand, if RBC ratio was increased in cell mixture, RBC depletion unit was saturated and the harmony between hydrodynamic and electrical conditions in the first part was damaged. This caused RBC trapping in the second unit decreasing MDR cancer cell selectivity of the system. This problem might be overcome by increasing the length of RBC depletion unit. 

### 3.3. Separation of Cells 

Separating and enriching a specific bioparticle from a heterogeneous mixture is a critical task that must be accomplished in many biological and clinical assays. As a label-free method, DEP depends on the inherent dielectric properties of biological particles determined by their structure and composition, making it a very attractive separation technique for BioMEMS applications. The DEP force exerted on particles in the non-uniform electric field varies for different particle types due to the existing differences between properties such as shape, size, and electrical characteristics. As a result of the differences in the dielectric polarizability of the particles, they show different DEP mobility in the non-uniform electric field, which enables selective separation and isolation with DEP. DEP-based separation has been applied extensively for the separation of many different types of bioparticles, including stem cells [110,189,190,191], circulating tumor cells (CTCs) [94,192], blood cells [193,194,195], bacteria [96,196,197,198], viruses [199,200], and DNA [201,202]. For instance, Song et al. reported a DEP-based continuous flow microfluidic device to separate human mesenchymal stem cells (hMSC) from their differentiation progenies (osteoblasts) [191]. DEP force generated with a large array of oblique interdigitated electrodes and the alternating field control was combined in the proposed device to attain continuous operation together with enabling improved collection efficiency and cell recovery. Collection efficiency up to 92% with purity up to 84% for hMSCs, and collection efficiency up to 67% with purity up to 87% for osteoblasts were obtained at the corresponding outlets. In another work, Yang et al. used a DEP-based microfluidic device to isolate human plasma from whole blood without applying any dilution or other preliminary handling procedure [195]. The separation was made possible by repelling and directing blood cells to the side channel by the nDEP force with the application of AC electric field. As a result, plasma was collected from the main channel outlet while blood cells were collected from the side channel outlet. They achieved plasma yield of 31% with a purity approaching 100%. Jones et al. utilized a microfluidic sorter device to achieve continuous separation of DNA molecules by size [203]. They showed tunable and selective separation of DNA molecules by size into different microchannel outlets by using AC insulator-based DEP (iDEP). The results revealed that over 90% sorting efficiency was achieved with the system presented. The initial study on DEP-based separation in our group was realized to accomplish direct injection of blood cells into microchannels and in-situ erythrocyte–leukocyte separation [204]. In the experiments, heparinized pure human blood droplets without any dilution were used and injection was performed on the chip without any external microfluidic connections to the device. Erythrocyte distillation from the normal human blood samples was accomplished with nDEP and cells enriched in leukocytes were directly injected into the collection reservoir.

DEP-based separation is widely applied for the separation of diseased cells to realize clinical applications in POC devices. Cells are widely used when examining disease prognosis because they are associated with the pathogenesis of many diseases, such as immune disorders, hematological malignancies, and cancer [205,206,207]. Diseased cells and cells at different proliferation or maturation stages have characteristic DEP responses as a result of variations in their cell state, internal and membrane structures, and morphologies. Therefore, DEP has great potential that allows diseased cells to be separated. DEP-based systems have successfully been applied to sort and isolate different diseased cell types, including, cancer cells, malaria infected cells, human African trypanosomiasis (HAT), dengue, and anthrax [103]. In particular, a great effort has been made to develop DEP-based cancer cell/CTC isolation systems due to the importance of cancer cell isolation in oncology and cancer disease diagnostics [105,208,209,210]. CTCs, present in the blood, are extremely rare cancer cells that detach from the primary tumor side and enter the peripheral bloodstream, move to distant regions by circulation and initiate metastasis by forming secondary tumors [211,212]. CTCs are strongly correlated with cancer invasiveness; therefore, their early detection and quantification carry significant information on monitoring metastatic progression, measuring treatment efficiency, and guiding the therapy [206,213,214,215]. As a part of completing the metastatic cascade successfully, the cell membrane of CTCs must have the ability to withstand hemodynamic forces and defeat the impacts of fluid shears present in the vessels, indicating that their surface morphology is more complex than normal peripheral blood cells [210,216]. Consequently, electrical properties, which depend on the morphological, structural, and chemical characteristics of the cells, are significantly different for CTCs and regular blood cells owing to the nature of the CTCs [154,208,217]. These distinct electrical properties that exist between CTCs and peripheral blood cells enable DEP to be a prominent isolation mechanism. Numerous studies showed that DEP-enabled devices have been successfully implemented to isolate CTCs [95,192,218,219,220,221]. Gupta et al. presented the Apostream™ system, the first commercial instrument for continuous flow enrichment of CTCs from peripheral blood cells through the use of DEP-field flow fractionation [100]. Experiments were performed with SKOV3 and MDA-MB-231 cancer cell lines spiked into approximately 12 × 10^6^ peripheral blood mononuclear cells obtained from 7.5 mL human donor blood sample after an initial Ficoll density gradient enrichment stage. They reported that the recovery rate was over 70% and viability was more than 97%. Recently reported studies have verified the ability to isolate CTCs from clinical samples using the ApoStream™ system [222,223]. 

**Figure 5:**
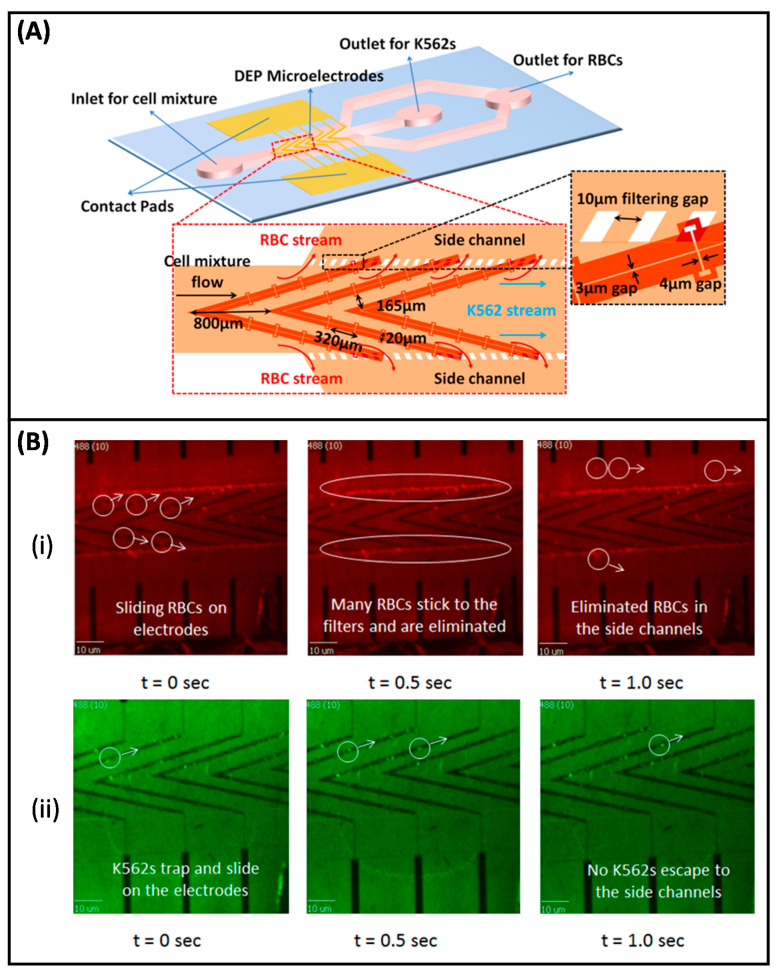
(**A**) Schematic of the device. (**B**) Experiments were performed with a flow rate of 30 µL/min at 20 V_pp_, 1 MHz. Manipulation of the RBCs stained with Cell Tracker Red (**i**), and K562 cancer cells stained with fluorescein diacetate (**ii**). All cells were directed to the side walls via pDEP by sliding over the electrodes. While the RBCs passed through the filters, the K562 cells moved through the main channel because they could not pass the filters [225]. Copyright 2017, Elsevier.

Considering the great potential of the DEP technique in CTC isolation, our group conducted studies in this area. Yılmaz et al. developed a microfabricated DEP-based device with spiral channel geometry to achieve high resolution cell separation [224]. In the proposed technique, spiral channels provided an efficient implementation of the long separation channels. The non-uniform electric field was created through concentric electrodes that provided electric field variation over radial distance. With the application of voltage, the cells initially placed in the central reservoir began to move along the spiral channel under the effect of the DEP force. At the end of successive turns in spiral channels, cells were separated according to their different CM factors. The proof-of-concept of the presented technique and implemented device was shown by conducting experiments using both beads with different sizes (1 µm and 10 µm) and K562 leukemia cells. In a later study, Özkayar integrated DEP and size-based filtration techniques into a single MEMS-enabled microfluidic chip to realize the enrichment of CTCs from blood with high throughput [225]. Hydrodynamic and pDEP forces were used to control the motion of cells over the planar electrodes used to create non-uniform electric field. The device was tested with RBCs and K562 cancer cells. The working principle of the device was based on filtering the cancer cells and directing them to the cancer cell outlet, while the smaller sized RBCs were removed from the main channel and directed to the waste outlet through the gaps (Figure 5). It was reported that the system can perform the enrichment operation at a high flow rate (30 µL/min). The results revealed that the RBCs were depleted by 60% at the cancer cell output, while 1.45-fold enrichment was obtained in K562 cells.

### 3.4. DEP for other Purposes 

In addition to the applications mentioned above, DEP, a stand-alone technique with significant advantages in particle manipulation, has been utilized as an important component in a number of biosensor applications, such as drug delivery [91], droplet [226,227], deposition, patterning, and tissue engineering [116,126,140,228,229,230,231,232], cytometry [233,234], collection, concentration, and trapping [235,236,237,238,239,240]. In cell patterning, which means arranging cells in desired patterns to mimic real tissue and to examine cell–cell interactions, the DEP method is used because it provides significant advantages, such as elimination of pre-modification processes, ease of handling, reduced cell damage, and precision [229,232]. Suzuki et al. described a method for patterning different types of cells via nDEP without applying any pretreatment of the culture slide [230]. Cells in the culture medium were aligned within 1 min under the effect of nDEP force generated with an interdigitated array electrode when an AC voltage was applied at 1 MHz, 12 V_pp_. Micropatterning of two cell types was achieved in 15 min with the presented system. This method allows conducting fundamental cell biology studies and analyzing cell–cell interactions between different cell types. One another application in this line of research is organ-on-a-chip (OOC) systems. OOC platform, combining tissue engineering and microfluidics, is a cell culture model developed to mimic cellular organization and interaction, physiological responses, functions and activities of tissues and organs [241,242,243,244]. OOC systems are powerful platforms for tissue analysis, disease modeling, toxicity screening, and new drug development that are critical for biological and pharmaceutical applications [245,246]. DEP has also been used to obtain complex cellular patterns in OOC applications [247,248]. For example, Schütte et al. developed a microfluidic system to study liver toxicity [248]. Prediction of liver toxicity poses a particularly significant challenge as liver cells grown in a cell culture experience a rapid loss of their liver specific functions. To address this need, they presented a microfluidic test system that was based on an organ-like liver 3D co-culture of hepatocytes and endothelial cells. The microfluidic system featuring of cell culture chambers with integrated electrodes enabled the assembly of primary human hepatocytes and endothelial cells in a sinusoidal-like manner with the use of DEP. 

Cytometry, which is a powerful method for analyzing particles by determining particle morphology, size, and chemical composition, is another field of bioapplication where DEP can be integrated. Yu et al. presented a microchannel to focus particles using DEP for cytometry applications [233]. DEP-based particle focusing was explored to replace the hydrodynamic focusing mechanism, one of the key components in cytometers. Experiments were performed with microbeads and human leukemia HL60 cells, and the results revealed that focusing operation of the cells was achieved using an AC voltage at frequencies below 100 kHz and voltage amplitude up to 15 V_pp_. This study proposed a portable system that could be integrated into biosensors and LOC devices and eliminated the use of sheath flow and fluid control systems which makes conventional cytometers complicated and bulky. In most biosensor platforms, insufficient molecular interaction problem is present due to the low concentration of the bioparticle of interest in the analyte. In this context, DEP is widely used in such systems to increase the local concentration, aggregation, or capture of target particles efficiently, in a short time, and with high precision. This allows performing consecutive operations such as identification, detection, measurement, and/or imaging on DEP-manipulated particles. Yang presented DEP integration into non-flow through biochip to assist immune-capture and detection of Salmonella Typhimurium, one of the major foodborne pathogenic bacteria [249]. In this study, two important functions of DEP to improve the performance of biosensors were demonstrated: (i) DEP provides an advantage in particle manipulation by concentrating cells from the suspension medium to different locations on the chip surface; (ii) DEP improves the efficiency of immune capture by bringing cells into close contact with antibodies immobilized at the chip surface. The device achieved considerably higher immuno-capture efficiencies with DEP (∼56.0% and ∼64.0% for 15 and 30 min with DEP, respectively); when compared with the immuno-capture efficiencies obtained without DEP (∼10.4% and ∼17.6% for 15 and 30 min without DEP, respectively). In another study, A549 human lung CTCs were detected by combining DEP and electrical impedance measurement using a single microfluidic device [235]. DEP manipulation was performed to direct the A549 cells and to trap them onto the desired sensing electrodes, resulting in a cell enrichment rate of over 90%. The target cells were concentrated by cell trapping electrodes and then identified by impedance responses. 

Nowadays, a great effort has been directed to develop LOC and biosensor platforms for viral diagnosis due to the massive viral outbreak of COVID-19. COVID-19, an infectious disease caused severe acute respiratory syndrome in humans, has resulted in more than 40.4 million infections and more than 1.1 million deaths by 21 October 2020 [250,251]. As in the case of some acute viral infections, a major contributor in the propagation of the COVID-19 disease is asymptomatic carriers, displaying no clinical symptoms [252,253]. Another concern is the low concentration of virus present in the samples, causing false negative results. DEP technique has provided promising results for the concentration and detection of viruses in biosensors and analytical tools [91,254,255,256,257,258]. Nakano et al. reported the DEP impedance measurement (DEPIM) method for the electrical detection of pathogenic viruses, namely, adenovirus, and rotavirus [255]. The presented DEPIM method consisted of two simultaneous processes which are DEP trapping of the target and measuring the impedance change, and the increase in conductivity with the number of trapped targets. Electrical detection of viruses in 60 s at concentrations as low as 70 ng/mL for adenovirus and 50 ng/mL for rotavirus was demonstrated. They also investigated the dielectric properties of the viruses in terms of their DEP behavior. In another attempt, Iswardy et al. proposed a microfluidic DEP chip to realize rapid detection of dengue virus (DENV) [256]. DEP force was used in this microfluidic chip to capture modified beads and the DENV modified with fluorescence label, as the detection target, was then captured on the modified beads by immunoreaction. The reusable platform (>50× reusable) allowed rapid on-chip detection (5 min) with a small required volume of DENV samples. 

**Figure 6:**
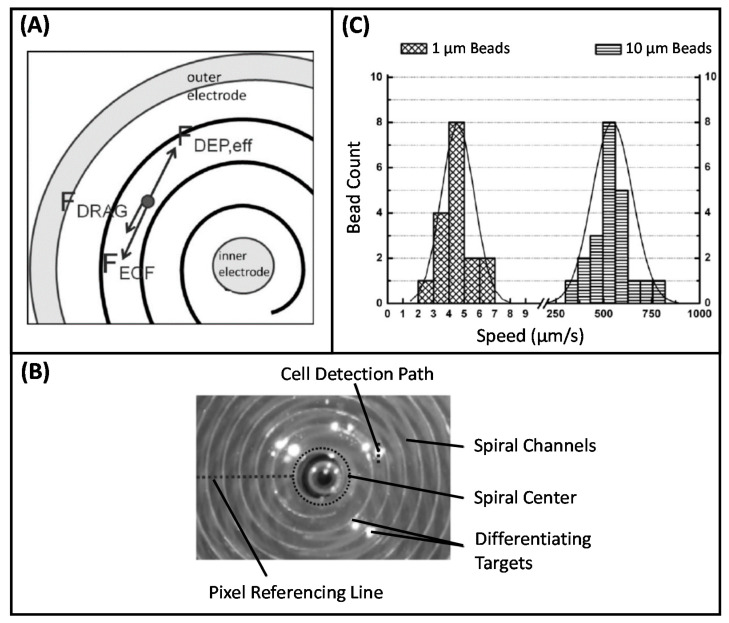
(**A**) Electrokinetic forces acting on the particles throughout their movement in the spiral channel. (**B**) Snapshot of recorded video processed using Image J to examine the speed histogram of 10 μm polystyrene particles. (**C**) Comparative histogram of extracted speeds of 1 and 10 μm beads. The number of beads is given with the data bars. The straight lines correspond to the best Gaussian fits. Results show that 1 µm and 10 µm particles have an average speed of 4.57 μm/s and 544 μm/s, respectively [259]. Copyright 2010, John Wiley and Sons.

As evidenced by studies in the literature, DEP assistance can be integrated into biochip and biosensor platforms to enhance device performance. In this context, our group has investigated the integration of DEP into biochip structures developed to perform different bioapplications. Yılmaz et al. presented a size quantization study based on a previously reported DEP-based device with spiral channel geometry [259]. The reported system works with standard laboratory instruments, facilitating the operation. Additionally, the device operated with a drop of sample, eliminating the need for external microfluidic connections. As explained previously, with the application of voltage coaxial electrode configuration generated a non-uniform electric field in the spiral microchannel. As a result of induced DEP force, particles initially placed in the central reservoir started to move along the channel. All electrokinetic forces acting on the particles through their motion in the channels were presented in Figure 6A. During this process, particles of different sizes began to separate after successive turns. The speed of the particles or the total distance traveled by the particles was related to their size and DEP characteristics (Figure 6B). Experiments were conducted with 1 µm and 10 µm polystyrene beads with a measured average speed of 4.57 μm/s and 544 μm/s, respectively (Figure 6C). The results indicated that the presented method could be applied to discrimination of particles with different radius, enabling the DEP-based chromatography system for cytometry applications.

**Figure 7:**
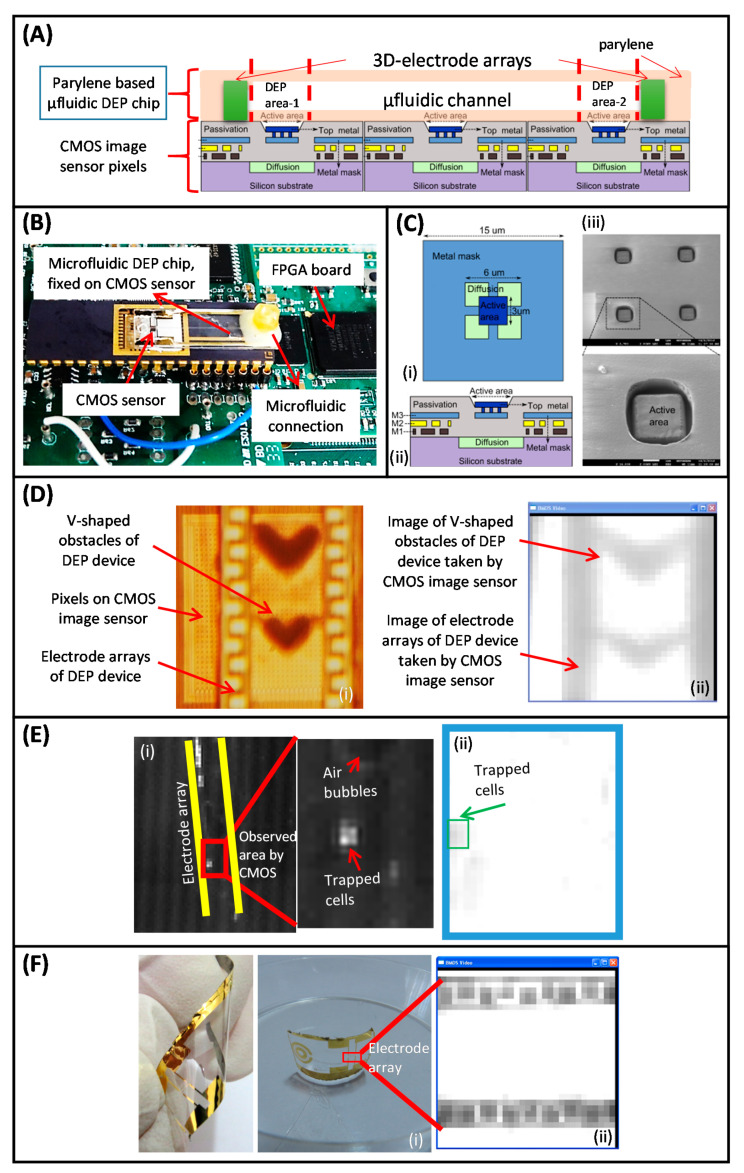
(**A**) The schematic illustration of the proposed lab-on-a-chip (LOC) system which integrates a microfluidic DEP chip on top of a CMOS image sensor. (**B**) The photograph of the integrated platform under test. (**C**) Pixel structure in CMOS image sensor with (**i**) its top view and (**ii**) cross-section illustrations, and (**iii**) SEM images. (**D**) View of DEP chip captured via (**i**) microscope, and (**ii**) CMOS sensor. (**E**) Images of trapped MDR K562 cells inside DEP device (f = 48.64 MHz, voltage = 9 V_pp_) captured via (**i**) fluorescence microscope, and (**ii**) CMOS sensor. (**F**) (**i**) Flexible parylene DEP device, and (**ii**) the view of the electrode array of it under CMOS imager [260]. Copyright 2015, IEEE.

In another study, Demircan et al. presented a fully integrated LOC system to accomplish label-free detection and real-time quantification of MDR K562 cells trapped with DEP [260]. In this study, a previously reported parylene-based MEMS fabricated microfluidic DEP chip was integrated on top of a CMOS image sensor for the first time (Figure 7A–C). The results showed that while MDR K562 cells were trapped under 9 V_pp_ at 48 MHz and a flow rate of 10 µL/min, wild type K562 cells were not trapped, confirming that the proposed DEP system could be used for MDR detection. The CMOS image sensor was able to quantify DEP trapped cells with a diameter as small as 3 μm with a noise level of 28.3 e^−^rms (Figure 7D,E). In addition, to enhance the image quality by improving focus and resolution of the CMOS images flexible-parylene DEP devices were also implemented (Figure 7F). Recently, this idea in which a DEP device integration on a CMOS image sensor was aimed at detecting and counting MCF7 human breast cancer cells [261]. A DEP device with interdigitated planar electrodes was implemented to realize trapping of MCF7 cells at 2 µL/min flow rate via 20 V_pp_, 1 MHz AC signal. Real-time imaging of the trapped cells in the DEP channel was carried out with a commercial CMOS sensor. An Android smartphone was integrated into the proposed system to acquire and process image data, and to supply power to the whole setup which eliminated the need for an external power supply. The cell count was performed automatically in the developed Android application and the counting results showed an average accuracy of 90%. As a result, the presented DEP-assisted LOC system allowed cells to be detected, visualized, and enumerated in label-free and lens-free manners.

## 4. Challenges, Conclusions, and Future Prospects 

DEP has been shown to have the potentials to be one of the most appropriate and assistive particle manipulation and analysis techniques in BioMEMS applications. The DEP manipulation of micro and nano bioparticles holds distinct promise as this technique depends on both cell dielectric properties and physiology. DEP is capable of realizing rapid manipulation and selective detection of bioparticles with high efficiency, accuracy, and sensitivity in a label-free manner. The DEP technique is highly compatible with the microfabricated biosensor and biochip platforms as well as microfluidics. This offers significant potential to replace bulky and complex instruments used in laboratories by developing suitable POC devices. DEP-based devices are cost- and time-efficient and user friendly which eliminates the need for highly trained personnel to perform the operation. In the research of medical science, DEP has been extensively employed to characterize, isolate, separate, sort, fractionate, concentrate, detect, collect, pattern, and trap various target bioparticles. 

Although the DEP technique provides a number of advantages and a broad range of bioapplications, there are still some challenges while developing successful DEP systems and their implementation into biosensor and POC platforms. One of the primary challenges is to develop an optimal electric field induced device design by considering the DEP response of the target particles to the electric field. This brings the requirement of accurate determination of dielectric properties and DEP responses of the target particles in designing DEP-based systems. As described in Section 3.1, ER and DEP spectrum methods are commonly used to determine the dielectric properties and/or DEP responses of particles. Although the dielectric properties of many different particle types have been determined in the literature, there exist some discrepancies between the data as a result of the dynamic nature of the cells together with the differences in measurement and preparation techniques followed. The dielectric properties of the particles show a significant dependence on the size, developmental stage, physiological state, and environmental factors. This causes alterations in their dielectric properties and in turn their DEP responses [179]. Consequently, the development of successful DEP-based particle manipulation systems requires the determination of the dielectric properties and DEP responses of the particles by carefully considering the state and environment of the cell. 

Another critical issue is the throughput of the DEP systems. Their throughput is lower compared to other manipulation techniques as a result of the limited range in which electric field gradients extend from insulating structures or electrodes. Considerable effort has been directed to increase the throughput of DEP-based devices. The primary strategies developed to overcome this limitation include new methods and materials, 3D electrode geometries, and appropriately designed architectures that improve the interaction between the particle and the large array of insulating structures or electrodes [102,262]. For example, increasing channel dimensions in trapping devices will result in an increase in throughput. However, this is not the case for continuous-flow devices. For devices with internal planar electrodes, microchannel height cannot be increased as the effective DEP force significantly reduces through the height of the channel. One alternative to solve this problem is employing 3D electrodes to exploit the *z*-axis. Wang et al. developed a separation strategy for lateral flow-through separation of particles in microfluidics by dual-frequency DEP with vertical interdigitated electrodes placed in the channel sidewalls [263]. Vertical interdigitated electrodes on the sidewall allowed the height of the microchannel to be increased without losing the electric field strength, unlike other multi-frequency DEP devices with planar electrodes. Consequently, high throughput sorting microfluidic devices could be achieved via DEP. Islam et al. presented a microfluidic device with 3D glass-like carbon microelectrodes to enrich yeast cells with a throughput of 10 µL/min [264]. In another study, Cheng et al. reported a microfluidic device with 3D DEP gates to perform continuous bioparticle filtering, sorting, and trapping [265]. Multiple particle types, latex particles, *Candida albicans*, *Lactobacillus*, and *Escherichia coli Nissle*, were sorted and concentrated with this system. The platform accomplished continuous operation with a throughput of 3 µL/min. Similarly, out-of-plane insulator arrays could be employed instead of in plane ones. In this concept, the electric field is effective along the *z*-axis rather than in the same plane as the operation (*x-y* plane). Čemažar et al. reported a contactless DEP microfluidic device in which the insulating pillars were on the same scale as the diameter of a typical cell (20 µm) [266]. The device was tested with a highly aggressive Mouse Ovarian Surface Epithelial (MOSE) cell line. The results of the proposed cell scale microstructures showed improved trapping efficiency, viability, and throughput. In this configuration, a high throughput of 2.2 mL/h was achieved while maintaining cells alive for further analysis. Although 3D microelectrodes and 3D microstructures have been used to increase the sorting or capturing efficiency of cells in DEP devices, their fabrication often requires conventional methods such as lithography and etching. These microfabrication methods could be complex and time consuming as they involve complicated instruments and multiple sequential processes. To address these issues, a new technique for creating liquid metal 3D microstructures was introduced by Tang et al. [267]. They formed 3D microstructures by fixing Galinstan microdroplets onto planar chromium/gold microelectrode pads by using DEP. They used these microstructures as 3D DEP microelectrodes for the trapping of tungsten trioxide (${\mathrm{WO}}_{3}$) nanoparticles flowing through a microfluidic channel. 

Despite improvements in the throughput, sample pretreatment, such as pre-enrichment or pre-focusing operations that can be done on-chip or off-chip, is essential in DEP-based systems for some specific applications, particularly isolation and detection of rare cells [262]. For example, the greatest technical challenge associated with CTC detection and isolation is the extremely low concentration of CTCs, compared with the significantly high number of blood cells (i.e., only few CTCs, 1-3000 CTCs per mL, present amongst 10^9^ RBCs per mL and 10^7^ leukocytes per mL) [268,269,270]. The integration of preprocessing operations into DEP-based systems has been implemented to overcome such challenges and to facilitate the manipulation of particles by making the numbers more manageable, as sample loss is intolerable. Moon et al. reported a microfluidic device that consists of serially integrated multi-orifice flow fractionation (MOFF) and DEP separation sections to separate human breast cancer cells (MCF7) from blood [219]. In this device, the hydrodynamic separation (primary) section achieved massive and rapid filtration and extraction of most blood cells via MOFF. After the MOFF section, DEP (secondary) section accomplished the separation of cancer cells from residual blood cells. The resultant MOFF–DEP combination achieved 162-fold enrichment of the MCF7 cells at a high flow rate (126 µL/min). The reported separation efficiencies for red and white blood cells were 99.24% and 94.23%, respectively. Pommer et al. achieved a size-based fractionation of blood and enriched platelets [271]. They developed a two-stage DEP activated cell sorter (DACS) device in which the whole blood sample was delivered to the system from the sample inlet and subsequently diluted with a buffer stream supplied from the buffer inlet. In this way, they achieved on-chip dilution. The DEP-based separation was conducted on the diluted blood sample and the fluid stream enriched with platelets were directed to the collection outlet.

One of the major problems arises from the fact that DEP manipulation of bioparticles is typically performed with low conductivity solutions while biological and physiological media have significantly higher conductivity [262]. DEP technique strongly depends on the conductivity of the buffer. When particles are suspended in a highly conductive buffer, pDEP is not effective and they tend to show nDEP response across the frequency spectrum [102]. High conductivity buffers can also lead to electrochemical reactions such as electrolysis at the edge of electrodes [101,135]. The Joule heating effect, excessive heat as a result of high conductivity, can cause severe cell damage, dehydration, and decreased device performance [91,101,228]. Consequently, it is hard to accomplish DEP-based manipulation in high conductivity physiological buffers. To avoid adverse effects of electrochemical reactions and Joule heating, and to reach more distinctive DEP responses, electrically low conductive buffers are mainly used in DEP systems [272,273]. However, this introduces a few concerns to DEP, regarding cell viability, alterations on cell functions and properties, and experimental time required to resuspension of particles in low conductivity buffer from their native media [139,274]. Working with low conductivity conditions limits the usage of DEP-based devices in real viable bioparticle separations. In order to avoid changes in dielectric properties of cells suspended in low conductivity buffers, DEP experiments should be completed in a short time [220,275]. In an attempt on this issue, Puttaswamy et al. accomplished patterning of human liver cells with nDEP by using microchip with titanium electrode arrays [276]. In this study, they investigated the effect of conductivity on cell adhesion and cell viability and proposed a low conductive buffer that improved cell viability to 2–3 h. This DEP buffer could be employed in other DEP-based studies where viability is a concern due to prolonged electric field exposure. In another study, to overcome problems associated with high conductivity medium in DEP operation, Park et al. reported a microfluidic device for continuous separation and concentration of target cells by directly processing physiological samples [277]. The device, a combination of nDEP separation channel and pDEP traps, was capable of directly processing samples by eliminating conductivity adjustments prior to sample introduction. They achieved separation and concentration of *E. coli* from either whole blood samples or human cerebrospinal fluid. 

Another critical challenge is the development of self-sufficient DEP systems to provide standalone and fully automated DEP-based POC devices or biosensor platforms. In these systems, the ultimate goal is to perform the intended analysis in a plug-and-play fashion by simply adding the sample, starting the process, and then viewing the results [278]. A complete self-powered and self-automated DEP system require integrating multiple steps such as sample preparation and handling, microelectrodes and microfluidics, detection, analysis, and display on a single platform [91]. As a result, system integration is one of the most critical issues in transferring DEP-based systems from laboratories to the clinical environment by installing portable and automated POC and biosensor platforms that eliminate the use of large and expensive devices, enable non-technical users to operate, reduce analysis time, and prevent sample loss [278,279]. A good attempt on this direction was reported by Gomez-Quiñones et al. who developed a sixteen-channel sinusoidal signal generator used as a stimulator for microfluidic devices focused on particle trapping, separation, and characterization through electrokinetic effects [280]. The stimulator was based on a CMOS circuit with a footprint of 1560 × 2030 µm, and an interface card that extended the voltage and frequency ranges and allowed the connection between the stimulator and the microfluidic chip. To verify the performance, the device was connected to a DEP-enabled microfluidic platform and successful DEP trapping experiments were performed with cells and polystyrene beads. The presented system eliminated the need for external signal generators and provided a standalone DEP platform in which only the power connection and user interface was needed. In another study, Qiao et al. developed a wirelessly powered microfluidic DEP device using printable RF circuit [281]. The device was formed from microfluidic structures and integrated RF circuit fabricated on a flexible plastic substrate using a roll-to-roll printing method. Electrical power at 13.56 MHz transmitted by a radio-frequency identification (RFID) reader is inductively coupled to the printed RFIC and converted into 10 V DC output, which provides sufficient power to drive a microfluidic device to manipulate biological particles such as beads and proteins via the DC–DEP. It was also stated that in the case of a bioelectronic device included in the system for cell impedance measurement, the obtained data and results could be wirelessly transmitted back to the RFID reader at 13.56 MHz through the printed antenna to realize wireless data transfer and storage. Overall, the presented wirelessly powered device removed entire wire connections and external devices from the system, presenting a significant advancement in the use of DEP-based systems as POC devices.

The field of MEMS-based biosensing and POC devices have attracted significant attention and shown huge growth since they provide rapid and accurate clinical outcomes regarding diagnosis and treatment, and eliminate the need for laboratories equipped with bulky instruments and trained operators. The biosensor and biochip platforms hold tremendous potential for the medical industry, healthcare, patient monitoring, and healthcare management. To realize successful POC devices in medicine and related applications, the manipulation of the bioparticles is one of the vital tasks. DEP has been proven to be a promising particle manipulation technique in numerous bioapplications such as characterization, isolation, concentration, and detection. The main goal for the coming years is to increase the validation and integration of DEP in clinical settings and POC devices. By striving for some of the mentioned challenges and requirements, along with innovations and developments, the DEP technique is expected to have a profound impact on a variety of biosensors and POC platforms.
